# On Poisons in Relation to Medical Jurisprudence and Medicine

**Published:** 1859-10

**Authors:** 


ART. VI.—*On Poisons in relation to Medical Jurisprudence and Medicine.* By Alfred Swaine Taylor, M. D., F. R. S., etc. Second American, from the second and revised London edition. Philadelphia: Blanchard & Lea, 1859. 8vo. pp. 755.

This volume is devoted to the consideration of the substances generally employed for murder and suicide. In these relations they are considered under the several points of view in which they are of interest and importance to practitioners of law and medicine. The progress of toxicology since the publication of the first edition, has required an entire remodeling of the subject. Many chapters have been struck out, and new chapters have been introduced, in order to fulfill the requirements of the present state of knowledge.

As an authority on the matters treated of, Dr. Taylor's work ranks second to none other. It claims a place in the library, not alone of the chemical analyst, but of every practicing physician, who is liable to be called on to investigate cases in which poisonous substances have been employed for the destruction of human life. The practitioner who regards his own reputation, as well as the interests of justice and humanity, will do well thus to provide for an emergency which may occur at any moment.

